# Comparison of Oral Microbe Quantities from Tongue Samples and Subgingival Pockets

**DOI:** 10.1155/2018/2048390

**Published:** 2018-04-26

**Authors:** André Göhler, Stefanie Samietz, Carsten Oliver Schmidt, Thomas Kocher, Ivo Steinmetz, Birte Holtfreter

**Affiliations:** ^1^Friedrich Loeffler Institute of Medical Microbiology, University Medicine Greifswald, Greifswald, Germany; ^2^Institute for Hygiene, Microbiology and Environmental Medicine, Medical University of Graz, Graz, Austria; ^3^Department of Prosthodontics, Gerostomatology and Biomaterials, University Medicine Greifswald, Greifswald, Germany; ^4^SHIP-Clinical-Epidemiological Research, Institute for Community Medicine, University Medicine Greifswald, Greifswald, Germany; ^5^Department of Restorative Dentistry, Periodontology, Endodontology, and Preventive and Pediatric Dentistry, University Medicine Greifswald, Greifswald, Germany

## Abstract

**Objectives:**

To improve understanding of periodontitis pathology, we need more profound knowledge of relative abundances of single prokaryotic species and colonization dynamics between habitats. Thus, we quantified oral microbes from two oral habitats to gain insights into colonization variability and correlation to the clinical periodontal status.

**Methods:**

We analyzed tongue scrapings and subgingival pocket samples from 237 subjects (35–54 years) with at least 10 teeth and no recent periodontal treatment from the 11-year follow-up of the Study of Health in Pomerania. Relative abundances of *Porphyromonas gingivalis*, *Aggregatibacter actinomycetemcomitans*, *Fusobacterium nucleatum*, *Streptococcus sanguinis*, total bacteria, and *Archaea* were correlated to clinically assessed pocket depths (PD) and clinical attachment levels (CAL).

**Results:**

Increased relative abundances of *P. gingivalis*, *A. actinomycetemcomitans*, and *F. nucleatum* were linked to increased levels of PD and CAL (i) on the subject level (mean PD, mean CAL) and (ii) in subgingival pockets. Relative abundances of *Archaea* from tongue samples correlated negatively with mean PD or mean CAL. Detection and quantity of bacterial species correlated weakly to moderately between the tongue and subgingival pocket, except for *Archaea*.

**Conclusions:**

Relative abundances of specific oral species correlated weakly to moderately between habitats. Single species, total bacteria, and *Archaea* were linked to clinically assessed severity of periodontitis in a habitat-dependent manner.

## 1. Introduction

Various microorganisms colonize oral habitats [1–4]. Each habitat appears to be preferentially populated by different and somewhat unique sets of microbes, whereas in periodontal disease the microbial profile in the subgingival pocket seems to narrow [5]. Periodontitis-associated microorganisms colonize not only subgingival pockets but also other habitats like the tongue dorsum. It harbors species such as *Fusobacterium* and *Porphyromonas* [6, 7] and further acts as a reservoir for recolonization of periodontal pockets after periodontal therapy [8, 9]. This raises the question whether the occurrence and proportion (relative abundance) of different taxa might affect the microbial interplay in the oral cavity. However, comparing the distribution and quantity of single prokaryotic species is complicated because of various detection and quantification methods [9–13]. In a recent study, we used qPCR for quantification of a single species [7], serving as representatives for different stages of oral biofilm formation [9, 14, 15].

Information about *Archaea* in context with periodontitis is rare. According to the published literature, they were detected in periodontally diseased but not in healthy gingival sulci [16–20]. In contrast, we detected archaeal 16S rRNA sequences from tongue scrapings in healthy and periodontally diseased subjects in a previous study [7]. However, other recent studies showed that methanogens were enriched in chronic periodontitis and drive periodontitis due to their metabolic capacity [21, 22].

While our previous study clearly demonstrated the advantage of using bacterial relative abundance levels, it did not compare bacterial colonization patterns between the tongue and the subgingival pocket habitat, considering also the clinical periodontal status [7]. Therefore, we aimed at describing the relation between the relative abundance of four oral microbes (*P. gingivalis*, *A.* actinomycetemcomitans, *F. nucleatum*, and *S. sanguinis*), total bacteria, and *Archaea* in two oral habitats (the tongue dorsum and subgingival pocket) and clinically assessed periodontitis measures in well-characterized study subjects from the 11-year follow-up of the population-based Study of Health in Pomerania (SHIP-2).

## 2. Materials and Methods

### 2.1. Study Population

The Study of Health in Pomerania (SHIP) is an ongoing longitudinal cohort study in North-East Germany. Baseline examinations (SHIP-0) were conducted between 1997 and 2001 with 4308 participants [23]. The 11-year follow-up (SHIP-2) included 2333 subjects (2008–2012).

For this study, we considered data of 1560 subjects, for whom data were available until the end of March 2011. Out of this collective, we selected individuals according to the following criteria: 35–54 years of age (*N*=402 excluded), no periodontal treatment within the last five years (*N*=116 excluded), complete dental status (*N*=31 excluded), at least 10 teeth (*N*=309 excluded), and complete information on further exclusion criteria. Pregnant women (*N*=1), subjects with diabetes mellitus and increased hemoglobin levels (HbA1c ≥ 6.5%) (*N*=14), and current smokers (*N*=115) were excluded, leaving 266 individuals. Of those, 237 individuals provided both tongue scrapings and subgingival pocket samples. The study protocol was a priori approved by the local Ethics Committee of the University of Greifswald (Registration Number BB 39/08a). This study was performed in accordance with “Strengthening the Reporting of Observational Studies in Epidemiology” guidelines for human research investigations.

### 2.2. Covariates

Sociodemographic and behavioral variables were assessed by computer-assisted personal interviews. School education was categorized as <10, 10, and >10 years. Smoking status was defined as never, former, and current smoking. We defined diabetes mellitus as a self-reported physician's diagnosis or intake of antidiabetic medication (Anatomical Therapeutic Chemical (ATC) A10). Body height and weight were determined using calibrated scales. The body mass index (BMI) was calculated and categorized as <25, 25–<30, and ≥30 kg/m^2^.

### 2.3. Dental Examination

Periodontal examinations comprised probing depths (PD) and clinical attachment levels (CAL). Measurements were assessed at four sites per tooth (disto/mid/mesiobuccal and midlingual) according to the half-mouth method, alternating on the left or right side, excluding third molars and using a periodontal probe (PCP-2, Hu-Friedy, USA). CAL was not measured if the determination of the cementoenamel junction was not clear. Teeth were counted excluding third molars. Five certified and licensed dentists conducted examinations. Calibration exercises were performed before and every 6–12 months during the course of the study. Intrarater correlations for CAL measurements were 0.67–0.89, and interrater correlation was 0.70.

### 2.4. Sample Collection and DNA Isolation

Tongue biofilm was taken from the middle third of the tongue dorsum with a sterile spatula [7]. The spatula was transferred into 2.0 ml of phosphate-buffered saline (PBS). After shaking vigorously for 30 s, the spatula was removed. Microbial suspensions in PBS were kept at −80°C until further processing. After supragingival plaque was removed with a cotton roll, subgingival plaque was collected from the mesiobuccal pocket of the most distally located, clinically examined, upper tooth in the periodontally examined quadrants. Paper points (ISO 35; Roeka, Langenau, Germany) were inserted until the pocket base for 10 seconds. To avoid cross contamination, the sampling site was confined with cotton rolls. Paper points were stored at −80°C. Before DNA extraction, 230 *µ*l of lysis buffer and 20 *µ*l of proteinase K (both MagNA Pure LC DNA Isolation Kit III, Roche, Mannheim, Germany) were added and samples were incubated at 65°C for 10 min and then at 95°C for 10 min. DNA was extracted as described elsewhere [7] and stored at −20°C.

### 2.5. Oligonucleotides, Plasmid Standards, and Quantitative PCR

Species- and domain-specific primers and probes (MWG-Eurofins, Ebersberg, Germany) applied in the qPCR assays are listed in Supplemental Table 1 and described in detail elsewhere [7]. Plasmid standards contained respective target sequences in a pSC-B-amp/kan vector backbone (Stratagene, La Jolla, USA). The terms “total bacteria” and “*Archaea*” describe the total number of detected bacterial species by using the primers/probes listed in Supplemental Table 1.

The selected oral microbes were quantified using an established qPCR assay [7]. All samples were analyzed in triplicates using a LightCycler 480-II, and *C*_t_ was calculated by ΔΔ*C*_t_ algorithm of the LightCycler 480 Software version 1.5.0 (Roche, Mannheim, Germany). Efficiencies between 1.9 and 2.1 ensured efficient qPCR amplification in all qPCR runs.

### 2.6. Statistical Analyses

Continuous data are shown as mean ± standard deviation (SD) if normally distributed (assessed by QQ plots) or median (25% quantile, 75% quantile) if nonnormally distributed. Categorical data are presented as numbers (percentages).

We calculated relative abundances for *P. gingivalis*, *F. nucleatum*, *A. actinomycetemcomitans*, and *S. sanguinis* by dividing the single species count by the number of 16S rRNA gene copies per sample. We knew that this method could possibly lead to an underestimation of single species relative abundances, because the estimation models for species counts within a sample are still controversially discussed [24, 25]. The sum score equals the sum of relative abundances for three (putatively) periodontal pathogens (*P. gingivalis*, *A. actinomycetemcomitans*, and *F. nucleatum*).

Relative abundance values were shifted by 1 and then log transformed (retaining distribution values of 0). To compare relative abundances from tongue scrapings across categories of mean PD/CAL, clinical variables were categorized (1st, 2nd + 3rd, and 4th quartile).

To show coherence between relative abundances and periodontal status, bacterial data from tongue samples and subgingival pocket samples were correlated to subject-level periodontal data (mean PD, mean CAL) and to site-specific levels of PD and CAL of the mesiobuccal pocket. To compare relative abundances across quartile-derived categories of mean PD and mean CAL, we applied Mann–Whitney *U* tests.

We used McNemar's test to determine differences in detection rates in tongue and subgingival pocket samples. To assess the magnitude to which reliability estimates might be biased, the Prevalence index (PI; (a − d)/*N*; range −1 to 1) and the Bias index (BI; (b − c)/*N*; range 0 to 1) were calculated. To circumvent problems associated with the use of kappa, the Prevalence and Bias Adjusted Kappa (PABAK) (2·*p*_o_−1; *p*_o_, observed agreement; range −1 to +1) was calculated [26] to determine the accordance in detection profiles between tongue scraping and the corresponding subgingival pocket sample (gold standard). Moreover, sensitivity and specificity with 95% confidence intervals were calculated. Relative abundances derived from tongue and subgingival pocket samples were correlated using Spearman's correlation coefficient (*r*_SP_; 95% confidence interval). All data analyses were performed using Stata/SE 12.0 [27].

## 3. Results

### 3.1. Study Population

Participants were 44.1 (SD 5.5) years old, and 42.2% were male (Table 1). Mean PD and mean CAL were 2.41 and 1.92 mm, respectively. The subgingival pocket sample originated from the second molar in 84.0%.

### 3.2. Correlation of Relative Abundances on the Tongue with Clinical Periodontal Status

Overall, correlations between relative abundances from tongue samples and clinical variables were weak (Table 2). Relative abundances of *P. gingivalis*, *A. actinomycetemcomitans*, *F. nucleatum*, and total bacteria were significantly related to mean CAL with Spearman's correlation coefficients ranging from *r*_sp_=−0.17 (95% CI: −0.29 to −0.04; *p*=0.009) for total bacteria to *r*_sp_=0.26 (95% CI: 0.14 to 0.38; *p* < 0.001) for *P. gingivalis*. Mean PD levels were weakly correlated to *P. gingivalis* (*r*_sp_=0.15; 95% CI: 0.03 to 0.28; *p*=0.02), *A. actinomycetemcomitans* (*r*_sp_=0.23; 95% CI: 0.11 to 0.35; *p* < 0.001), and *F. nucleatum* (*r*_sp_=0.25; 95% CI: 0.13 to 0.37; *p* < 0.001).

Relative abundances from tongue samples were unequally distributed across categories of mean PD and mean CAL (Table 3). Relative abundances of *P. gingivalis* (*p*=0.02), *A. actinomycetemcomitans* (*p*=0.0005), *F. nucleatum* (*p*=0.0002), and *Archaea* (*p*=0.04), % *Archaea* (*p*=0.006), and the sum score (*p*=0.001) differed significantly between the 1st and the 4th quartiles of mean PD. Relative abundances of *P. gingivalis* (*p*=0.0001), *A. actinomycetemcomitans* (*p*=0.005), *F. nucleatum* (*p*=0.005), and total bacteria (*p*=0.04) and the sum score (*p*=0.0001) differed significantly between the 1st and the 4th quartiles of mean CAL.

### 3.3. Correlation of Relative Abundances in Pocket Samples with Clinical Periodontal Status

For subgingival pocket samples, relative abundances correlated weakly to CAL and PD levels of respective mesiobuccal sites (Table 4). Except for *S. sanguinis*, all relative abundances showed weak, though significant, correlations above 0.1 with the mesiobuccal CAL (range 0.15–0.26). Mesiobuccal PD was weakly correlated to *P. gingivalis* relative abundance (*r*_sp_=0.18; 95% CI: 0.05 to 0.30; *p*=0.006).

**Figure 1:**
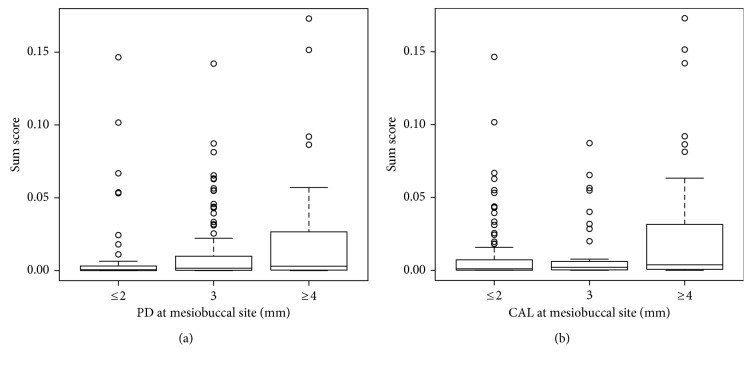
Distribution of the sum score (summed relative abundances of *P. gingivalis*, *A. actinomycetemcomitans*, and *F. nucleatum*) according to probing depth (PD (a)) or clinical attachment level (CAL (b)) at the same mesiobuccal sites.

Consistently, the sum score differed significantly comparing pockets with a mesiobuccal PD of ≤2 mm with those with a mesiobuccal PD of ≥4 mm (*p*=0.03; Figure 1). Similar findings were found for detections rates (*p*=0.046; Supplemental Figure 1) and relative abundances of *P. gingivalis* (*p*=0.01) with highest levels found in pockets with a mesiobuccal PD ≥ 4 mm (median relative abundance 6.3 × 10^−6^ (0; 1.3 × 10^−3^); Supplemental Table 3). Across categories of mesiobuccal CAL, significant differences in detection rates were found for *P. gingivalis* (*p*=0.03) and *A. actinomycetemcomitans* (*p*=0.046). Accordingly, differences in relative abundances were detected for all single species (*p* < 0.05) except *S. sanguinis*, *Archaea*, total bacteria, and the sum score (Supplemental Table 3).

### 3.4. Detection of Microorganisms in Tongue and Subgingival Pocket Samples

For *P. gingivalis* agreement between the tongue sample and the subgingival pocket sample was 78.5% with a moderate PABAK of 0.570 (Table 5). Among subjects with *P. gingivalis* negative subgingival pocket samples, 89.6% had a negative tongue sample (specificity). Among subjects with *P. gingivalis* positive subgingival pocket samples, 61.3% had a positive tongue sample (sensitivity). *F. nucleatum* was detected three times less often in tongue samples (*N*=53) compared to subgingival pocket samples (*N*=168). *S. sanguinis* was detected similarly often in both habitats with a high agreement rate (78.5%) and a high specificity (77.7%) and sensitivity (80.9%). For *Archaea*, agreement was 32.1% and PABAK was low (−0.359). Archaeal sequences were found four times more often in tongue samples (*N*=173) compared to subgingival pocket samples (*N*=40).

It must be mentioned that the PI and the BI were strongly deviating from the Null for *A. actinomycetemcomitans* (PI = 0.565), *F. nucleatum* (BI = 0.485), and *Archaea* (BI = −0.561), indicating some degree of bias, that would have distracted the kappa. Here, the PABAK provides an unbiased estimate of reliability.

Altogether, 16.1% of tongue scrapings but 43.1% of subgingival pocket samples contained at least two of the three (putative) periodontal pathogens (*P. gingivalis*, *A. actinomycetemcomitans*, or *F. nucleatum*). Accordingly, the number of detected pathogens from tongue and subgingival pocket samples correlated weakly (*r*_sp_=0.35; 95% CI: 0.24 to 0.46).

### 3.5. Correlation between Relative Abundances from Tongue and Subgingival Pocket Samples

Single species and total bacterial relative abundances demonstrated weak to moderate correlations between both habitats (Table 6). Correlations were moderate for relative abundances of *P. gingivalis* (*r*_sp_=0.63; 95% CI: 0.55 to 0.70; *p* < 0.001) and *S. sanguinis* (*r*_sp_=0.56; 95% CI: 0.46 to 0.64; *p* < 0.001) and weak for relative abundances of *A. actinomycetemcomitans* (*r*_sp_=0.16; 95% CI: 0.03 to 0.28; *p*=0.02), *F. nucleatum* (*r*_sp_=0.21; 95% CI: 0.09 to 0.33; *p*=0.001), and total bacteria (*r*_sp_=−0.14; 95% CI: −0.26 to 0.01; *p*=0.03). For illustration purposes, we present untransformed relative abundances in Supplemental Table 2. Restricting samples to those with positive relative abundances from tongue scrapings and subgingival pocket samples lead to more than halved correlation coefficients for single species (Supplemental Table 2).

## 4. Discussion

To elucidate the interplay of prokaryotic colonization in two habitats, we analyzed tongue and subgingival pocket samples from 237 participants of the 11-year follow-up of the population-based SHIP by qPCR. Relative abundances of *P. gingivalis*, *A. actinomycetemcomitans*, and *F. nucleatum* from subgingival pocket samples and tongue samples were linked to corresponding levels of PD and CAL. Quantitative inspection of oral microbes showed weak to moderate correlations in detection and quantity of oral prokaryotes between the tongue and subgingival pocket, though to different degrees depending on the species.

*P. gingivalis* is commonly regarded as periodontitis related [28, 29]. Forty-six percent of our participants were *P. gingivalis* positive on the tongue and/or in the subgingival pocket, with a significantly higher detection rate (*p*=0.003) in the periodontal pocket (39.2%) compared to the tongue (30.4%). For the tongue, this rate was lower than previously described (56.7%) [7]. Furthermore, relative abundances of *P. gingivalis* were significantly higher in subgingival pocket samples compared to tongue samples (Table 6). Further, relative abundances of *P. gingivalis* increased with increasing periodontal breakdown (Supplemental Table 3). In our study, *P. gingivalis* made up to 0.071% of total bacteria (relative abundance) in the subgingival plaque of *P. gingivalis* positive samples (*N*=108). In addition, the strong correlation of the relative abundance between tongue and subgingival samples (*r*_SP_=0.63; 95% CI: 0.55 to 0.70) confirms an interaction of these two habitats [30]. Overall, the effect of *P. gingivalis* alone was not prominent; this suggests that an ensemble of different bacteria in the respective oral habitat may determine the periodontal disease severity. However, it is possible that some taxonomic entities (sometimes referred to as “keystone pathogens”) at a specific metabolic state play a more prominent role in promoting inflammation [22, 31].

*A. actinomycetemcomitans* is often found in association with periodontitis. Compared to our previous study (46.2%) [7], the overall detection rate of *A. actinomycetemcomitans* was lower (37.1%). One explanation may be the selection process: instead of selecting healthy and periodontally diseased pairs, we selected all subjects fulfilling the inclusion criteria. Thus, we might have selected more subjects with mild or moderate periodontitis compared to the previous study. Furthermore, the tongue was significantly (*p* < 0.001) less often colonized with *A. actinomycetemcomitans* (14%) compared to the subgingival pocket (29.1%). In Swiss adolescents, this bacterium was detected with similar rates on the tongue (approximately 20%) and with lower rates for shallow pockets (approximately 15–17%) [30]. In a Swedish study, similar detection rates of 30% were found in subgingival plaques of periodontitis patients [32]. In this study, relative abundances on the tongue were only weakly associated with the periodontal status, which is in contrast to findings from the previous study [7]. Taken together, these observations strengthen the hypothesis that tongue levels of *A. actinomycetemcomitans* are only weakly associated with severity of chronic periodontitis.

*F. nucleatum*, which bridges early-colonizing with late-colonizing pathogens, was found in 74.7% of all tested subjects. The detection rate was approximately three times lower in tongue samples compared to subgingival pocket samples. In line with this, we found higher relative abundances in the later ones. Relative abundances of *F. nucleatum* on the tongue and in the subgingival pocket correlated weakly with corresponding levels of PD and CAL. However, we might assume that members of the genus *Fusobacterium* belong to the core oral human microbiome [10, 33]. The sum score was a useful marker of chronic periodontitis since higher levels correlated with higher levels of PD and CAL in both habitats (Figure 1).

*S. sanguinis* frequently colonizes in the healthy human mouth [34, 35]. In contrast to another study in which *S. sanguinis* was not detected on the tongue dorsum of any of the five examined healthy subjects [3], we detected *S. sanguinis* on the middle third of the tongue dorsum in 44.7% of subjects. However, this rate was lower compared to the detection rate of 57.4% in our previous study [7]. In subgingival pocket samples, we detected *S. sanguinis* in 37.5% of samples. This value was between the reported carriage percentages of 25% (periodontally diseased) and 40% (healthy) of a Japanese study [33]. Moreover, the proportion of *S. sanguinis* in positive subgingival plaques was 0.37%, which was in line with a previous study reporting a proportion of 0.33% ± 0.57 for *S. sanguinis* in healthy subjects [33].

Members of the *Archaea* also colonize the oral cavity, especially within subgingival plaques and on tooth surfaces [21, 36–38], while the diversity seems to be very narrow. We detected archaeal sequences in 73.0% of tongue samples, but only 16.9% of subgingival pocket samples. The latter proportion was lower than the so far reported detection range for subgingival pocket samples of 36% [39] to 96.4% [36]. However, the archaeal abundance is probably higher in periodontal pockets with >5 mm pocket depth [21], linking the archaeal abundance to more severe periodontal disease. In this study, we did evaluate a relation between archaeal load and severe probing depths, because the number of subjects with mesiobuccal PD > 5 mm was only seven, precluding further analyses.

Further, Matarazzo et al. found strong correlations between *P. gingivalis* and the archaeal count (*r*=0.75) in subjects with chronic periodontitis [40]. We found moderate correlations between total bacterial and archaeal load on the tongue (*r*_sp_=0.53; 95% CI: 0.44 to 0.62) and within the subgingival plaque (*r*_sp_=0.41; 95% CI: 0.30 to 0.51). The relative abundance of *F. nucleatum* was only weakly related to the archaeal relative abundance in subgingival plaque (*r*_sp_=0.14; 95% CI: 0.02 to 0.27). Thus, our study only slightly supports the hypothesis of Matarazzo et al. that *Archaea* may favor the settlement of some anaerobic bacterial species such as *P. gingivalis*, *F. nucleatum*, or *Prevotella intermedia* [21, 40]. Very weak and nonsignificant correlations were found between relative abundances of *P. gingivalis* or *A. actinomycetemcomitans* and archaeal load in both habitats (−0.08 < *r*_SP_ < 0.09) showing that not all virulent species are related to the archaeal load. Additionally, archaeal relative abundances on the dorsal tip of the tongue and the subgingival pocket did not correlate (*r*_SP_=−0.10; 95% CI: −0.23 to 0.03). This, together with the previous observation that low archaeal relative abundances correlate with health-associated effects [7] indicates a more complex picture. Further, metagenome analysis might reveal distinct bacterial/archaeal patterns, which dominate in each habitat depending on the periodontal condition.

For this study a well-defined, homogenous, and periodontally untreated subgroup was a priori selected to ensure that associations seen between tongue scrapings, pocket samples, and clinical periodontal status are more likely to be unbiased by subject-related factors. Specifically, with regard to disease severity, study subjects cover the range from periodontally healthy to moderately diseased subjects, reflecting the general population in contrast to clinical patient cohorts. Some limitations deserve consideration. First, periodontal examinations were taken according to the half-mouth method at four sites per tooth, which might have led to an underestimation of periodontal disease severity on the subject level [41]. Second, an aspect common to most sampling methods applied in oral habitats is that cross contamination of samples cannot be completely excluded.

## 5. Conclusions

In this study, relative abundances of specific periopathogenic bacteria sampled from subgingival pocket samples and tongue samples correlated significantly to corresponding levels of PD and CAL. Further, we indicated relevant correlations for four periodontal bacteria and *Archaea* between a global oral habitat (the tongue) and a local site (the subgingival pocket sample). This strengthens the hypothesis that the tongue could serve as a reservoir for oral bacteria. In consequence, considering the relation between both habitats might be helpful during/after dental cleaning-events or reinfections. Moreover, the detection and quantification of tongue microbes might help to determine the risk of recolonization of debrided sites after periodontal treatment. Further, the strong correlations between *P. gingivalis* and periodontal parameter variables point to its usage as an indicator for periodontal disease severity.

## Figures and Tables

**Table 1:** Characteristics of study subjects (*N*=237).

	Mean ± SD or number (%)
Age, years	44.1 ± 5.5
Males	100 (42.2%)
School education	
<10 years	5 (2.1%)
10 years	169 (71.3%)
>10 years	63 (26.6%)
Smoking status	
Never smokers	128 (54.0%)
Former smokers	109 (46.0%)
Body mass index, kg/m^2^	
<25	90 (38.0%)
25–<30	97 (40.9%)
≥30	50 (21.1%)
Information on periodontal status on the subject level	
Bleeding on probing, %	20.5 ± 18.1
Mean PD, mm	2.41 ± 0.36
Percentage of sites with PD ≥ 4 mm, %	8.0 ± 10.4
Mean CAL, mm	1.92 ± 0.91
Percentage of sites with CAL ≥ 4 mm, %	11.3 ± 16.3
Tooth count (excluding third molars)	24.8 ± 3.2
Information on-site level	
Tooth position	
3	2 (0.8%)
4	4 (1.7%)
5	10 (4.2%)
6	22 (9.3%)
7	199 (84.0%)
PD, mm (*N*=232)	
1-2	46 (19.8%)
3	130 (56.0%)
≥4	56 (24.2%)
CAL, mm (*N*=186)	
0–2	100 (53.8%)
3	38 (20.4%)
≥4	48 (25.8%)

Data are presented as mean ± standard deviation (SD) or numbers (percentages); PD, probing depth; CAL, clinical attachment level.

**Table 2:** Spearman's correlation coefficients (*r*_SP_; 95% confidence intervals) between clinical periodontal variables and relative abundances from tongue scrapings (on the subject level).

*r*_SP_ (95% CI)	Tongue scraping	
	Mean CAL (*N*=237)	Mean PD (*N*=237)
*P. gingivalis*	0.26 (0.14, 0.38)	0.15 (0.03, 0.28)
*A. actinomycetemcomitans*	0.24 (0.12, 0.36)	0.23 (0.11, 0.35)
*F. nucleatum*	0.23 (0.11, 0.35)	0.25 (0.13, 0.37)
*S. sanguinis*	−0.01 (−0.14, 0.11)	−0.001 (−0.13, 0.13)
*Archaea*	−0.11 (−0.24, 0.01)	−0.11 (−0.23, 0.02)
% *Archaea*	−0.10 (−0.23, 0.03)	−0.15 (−0.27,−0.02)
Total bacteria	−0.17 (−0.29, −0.04)	−0.08 (−0.20, 0.05)

PD, probing depth; CAL, clinical attachment level; *r*_SP_, Spearman's correlation coefficient; 95% CI, 95% confidence interval.

**Table 3:** Levels (median with Q25% and Q75%) of bacterial relative abundances (log10 transformed) from tongue scrapings across categories of mean probing depths (PD) or mean clinical attachment levels (CAL).

	Mean PD			Mean CAL		
	1.56–2.18 mm (*n*=59)	2.18–2.61 mm (*n*=120)	2.61–3.91 mm (*n*=58)	0.13–1.23 mm (*n*=60)	1.23–2.54 mm (*n*=118)	2.54–4.45 mm (*n*=59)
*P. gingivalis*	0 (0, 5.1·10^−6^)	0 (0, 8.6·10^−7^)	0 (0, 2.7·10^−5^)^∗∗^	0 (0, 0)	0 (0, 5.8·10^−6^)^∗^	0 (0, 3.3·10^−5^)^∗,∗∗^
*A. actinomycetemcomitans*	0 (0, 0)	0 (0, 0)^∗^	0 (0, 1.3·10^−7^)^∗,∗∗^	0 (0, 0)	0 (0, 0)	0 (0, 1.5·10^−8^)^∗,∗∗^
*F. nucleatum*	0 (0, 0)	0 (0, 0)^∗^	0 (0, 1.6·10^−5^)^∗^	0 (0, 0)	0 (0, 0)	0 (0, 1.4·10^−5^)^∗,∗∗^
*S. sanguinis*	0 (0, 5.2·10^−7^)	0 (0, 2.3·10^−6^)	0 (0, 1.6·10^−6^)	0 (0, 1.5·10^−6^)	0 (0, 1.7·10^−6^)	0 (0, 2.1·10^−6^)
*Archaea*	2.67 (2.52, 2.80)	2.70 (1.09, 2.84)	2.42 (0.00, 2.79)^∗,∗∗^	2.71 (2.54, 2.83)	2.61 (0.00, 2.80)	2.59 (0.00, 2.87)
% *Archaea*	0.015 (0.008, 0.024)	0.012 (0.002, 0.029)	0.007 (0, 0.019)^∗,∗∗^	0.015 (0.008, 0.027)	0.010 (0, 0.023)	0.010 (0, 0.035)
Total bacteria	6.41 (6.24, 6.63)	6.48 (6.16, 6.74)	6.32 (6.10, 6.70)	6.48 (6.25, 6.66)	6.41 (6.19, 6.72)	6.30 (6.02, 6.66)^∗^
Sum score	0 (0, 7.0·10^−6^)	0 (0, 9.6·10^−6^)	1.0·10^−5^ (0, 5.5·10^−5^)^∗,∗∗^	0 (0, 7.0·10^−7^)	1.2·10^−7^ (0, 1.3·10^−5^)^∗^	1.3·10^−5^ (0, 5.9·10^−5^)^∗,∗∗^

The sum score was defined as the sum of relative abundances for *P. gingivalis* + *A. actinomycetemcomitans* + *F. nucleatum*; groups were determined as follows: 1st quartile, 2nd + 3rd quartile, and 4th quartile; ^∗^ *p* < 0.05 in Mann–Whitney *U* tests versus 1st quartile; ^∗∗^ *p* < 0.05 in Mann–Whitney *U* tests versus (2nd + 3rd) quartile.

**Table 4:** Spearman's correlation coefficients (*r*_SP_; 95% confidence intervals) between clinical periodontal variables at the mesiobuccal site and relative abundances from subgingival pockets (site level).

*r*_SP_ (95% CI)	Subgingival plaque	
	Mesiobuccal CAL (*N*=186)	Mesiobuccal PD (*N*=232)
*P. gingivalis*	0.26 (0.12, 0.39)	0.18 (0.05, 0.30)
*A. actinomycetemcomitans*	0.18 (0.03, 0.31)	0.10 (−0.03, 0.22)
*F. nucleatum*	0.23 (0.09, 0.36)	0.07 (−0.06, 0.20)
*S. sanguinis*	0.07 (−0.08, 0.21)	−0.07 (−0.19, 0.06)
*Archaea*	0.18 (0.04, 0.31)	0.05 (−0.08, 0.18)
% *Archaea*	0.17 (0.02, 0.30)	0.04 (−0.09, 0.17)
Total bacteria	0.15 (0.01, 0.29)	0.12 (−0.01, 0.24)

PD, probing depth; CAL, clinical attachment level; *r*_SP_, Spearman's correlation coefficient; 95% CI, 95% confidence interval.

**Table 5:** Cross table for the detection (no/yes) of the microorganisms in tongue scrapings and subgingival samples. Additionally, percentages of agreement, Prevalence and Bias Adjusted Kappa (PABAK), specificities, and sensitivities are given.

Tongue scraping	Subgingival sample		Sum	P^∗^	Agreement	PABAK	Specificity(95% CI)	Sensitivity(95% CI)
	No detection	Detection						
*P. gingivalis*								
No detection	129 (89.6%)	36 (38.7%)	165	<0.001	78.5%	0.570	89.6%(83.4 to 94.1%)	61.3%(50.6 to 71.2%)
Detection	15 (10.4%)	57 (61.3%)	72					
Sum	144	93	237					

*A. actinomycetemcomitans*								
No detection	149 (88.7%)	54 (78.3%)	203	0.04	69.2%	0.384	88.7%(82.9 to 93.1%)	21.7%(12.7 to 33.3%)
Detection	19 (11.3%)	15 (21.7%)	34					
Sum	168	69	237					

*F. nucleatum*								
No detection	60 (87.0%)	124 (73.8%)	184	0.03	43.9%	−0.122	87.0%(76.7 to 93.9%)	26.2%(19.7 to 33.5%)
Detection	9 (13.0%)	44 (26.2%)	53					
Sum	69	168	237					

*S. sanguinis*								
No detection	114 (77.0%)	17 (19.1%)	131	<0.001	78.5%	0.570	77.7%(69.4 to 83.5%)	80.9%(71.2 to 88.5%)
Detection	34 (23.0%)	72 (80.9%)	106					
Sum	148	89	237					

*Archaea*								
No detection	50 (25.4%)	14 (35.0%)	64	0.21	32.1%	−0.359	25.4%(19.5 to 32.1%)	65.0%(48.3 to 79.4%)
Detection	147 (74.6%)	26 (65.0%)	173					
Sum	197	40	237					

^∗^McNemar test; 95% CI, 95% confidence interval; PABAK, Prevalence and Bias Adjusted Kappa.

**Table 6:** Overview on relative abundances (log10 transformed) for tongue scrapings and subgingival samples and respective Spearman's correlation coefficients (with 95% confidence intervals).

	Relative abundances		*r*_SP_ (95% CI)
	Tongue scraping	Subgingival sample	
*P. gingivalis*	0 (0, 6.3·10^−6^)	0 (0, 8.1·10^−5^)	0.63 (0.55, 0.70)
*A. actinomycetemcomitans*	0 (0, 0)	0 (0, 3.7·10^−5^)	0.16 (0.03, 0.28)
*F. nucleatum*	0 (0, 0)	8.6·10^−4^ (0, 5.8·10^−3^)	0.21 (0.09, 0.33)
*S. sanguinis*	0 (0, 1.6·10^−6^)	0 (0, 8.9·10^−4^)	0.56 (0.46, 0.64)
*Archaea*	2.63 (0, 2.82)	0 (0, 0)	−0.10 (−0.23, 0.03)
% *Archaea*	0.011 (0, 0.025)	0 (0, 0)	−0.01 (−0.14, 0.12)
Total bacteria	6.41 (6.16, 6.69)	4.22 (3.99, 4.47)	−0.14 (−0.26, 0.01)

Relative abundances are given as median (Q25%, Q75%); *r*_SP_, Spearman's correlation coefficient; 95% CI, 95% confidence interval.

## Supplementary Materials

Supplementary Materials Supplemental Table 1: sequence information, specificity, amplicon size, and molarity of oligonucleotide primers and probes used in the qPCR assays. Supplemental Table 2: correlation of abundances between tongue scrapings and subgingival samples. Supplemental Table 3: median relative abundances (log10 transformed; with 25% and 75% quantiles) from subgingival pocket samples according to categories of mesiobuccal probing depths and clinical attachment levels (categorized as ≤2, 3, and ≥4 mm). Supplemental Figure 1: detection rates of bacteria in subgingival plaques according to (a) mesiobuccal probing depth (PD) or to (b) mesiobuccal clinical attachment level (CAL). *P* values were calculated from chi-squared tests evaluating differences in detection rates among groups.
