# Erratum to “Comparison of Oral Microbe Quantities from Tongue Samples and Subgingival Pockets”

**DOI:** 10.1155/2018/5615780

**Published:** 2018-07-19

**Authors:** André Göhler, Stefanie Samietz, Carsten Oliver Schmidt, Thomas Kocher, Ivo Steinmetz, Birte Holtfreter

**Affiliations:** ^1^Friedrich Loeffler Institute of Medical Microbiology, University Medicine Greifswald, Greifswald, Germany; ^2^Institute for Hygiene, Microbiology and Environmental Medicine, Medical University of Graz, Graz, Austria; ^3^Department of Prosthodontics, Gerostomatology and Biomaterials, University Medicine Greifswald, Greifswald, Germany; ^4^SHIP-Clinical-Epidemiological Research, Institute for Community Medicine, University Medicine Greifswald, Greifswald, Germany; ^5^Department of Restorative Dentistry, Periodontology, Endodontology, and Preventive and Pediatric Dentistry, University Medicine Greifswald, Greifswald, Germany

In the article titled “Comparison of Oral Microbe Quantities from Tongue Samples and Subgingival Pockets” [1], there was an error in the Acknowledgments, which should be corrected as follows.
