# Supplementary material for: Comparison of Oral Microbe Quantities from Tongue Samples and Subgingival Pockets
Source: Int J Dent. 2018 Apr 26;2018:2048390. doi: 10.1155/2018/2048390 (PMC5944217; doi:10.1155/2018/2048390)
Supplement: Supplementary Materials — Supplemental Table 1: sequence information, specificity, amplicon size, and molarity of oligonucleotide primers and probes used in the qPCR assays. Supplemental Table 2: correlation of abundances between tongue scrapings and subgingival samples. Supplemental Table 3: median relative abundances (log10 transformed; with 25% and 75% quantiles) from subgingival pocket samples according to categories of mesiobuccal probing depths and clinical attachment levels (categorized as ≤2, 3, and ≥4 mm). Supplemental Figure 1: detection rates of bacteria in subgingival plaques according to (a) mesiobuccal probing depth (PD) or to (b) mesiobuccal clinical attachment level (CAL). P values were calculated from chi-squared tests evaluating differences in detection rates among groups. [file 2048390.f1.pdf]

SUPPLEMENTARY MATERIAL

Supplemental Table 1. Sequence information, specificity, amplicon size, and molarity of oligonucleotide primers and probes used in the qPCR assays.

Taxon	Oligonucleotide sequence (5' -> 3') [a]	Target gene; detection limit and amplicon size	Reference	Molarity
Porphyromonas gingivalis	F: AGGATCGCTCAGCGTAGCATT	Arg-gingipain	[1]	0.10 µM
	R: CCTACGTGTACGGACAGAGCTATA	(Rgp), (10 copies/PCR), 71 bp	[1]	0.50 µM
	P: FAM-TCGCCCGGGAAGAACTTGTCTTCA-BHQ1		[1]	0.10 µM
Aggregatibacter actinomycetemcomitans	F: ACGCAGACGATTAACTGAATTTAA	Leukotoxin C	[2]	0.10 µM
	R: GATCTTCACAGCTATATGGCAGCTA	(*lktC*), (10 copies/PCR), 77 bp	[1]	0.25 µM
	P: FAM-TTACCCTTCTACCGTTGCCATGGG-BHQ1		[2]	0.10 µM
Fusobacterium nucleatum	F: TGGCATAGCTTCACCTTTGA	β-subunit of RNA polymerase	[2]	0.25 µM
	R: CAAAGACTTGGGGAAATGGA	(*rpoB*), (10 copies/PCR), 144 bp	[2]	0.50 µM
	P: FAM-TGCTCCATAAGCTTCCAATGCCCA-BHQ1		[2]	0.10 µM
Streptococcus sanguinis	F: GGCGCCTGTTAATACTGAGC	Glycosyl transferase P	[3]	0.10 µM
	R: GTTTTTCCATCCTTGAGGATAGC	(*gtfP*), (10 copies/PCR), 330 bp	[3]	0.50 µM
	P: FAM-TCGATGCAGAGACCGGAGCC-BHQ1		[2]	0.10 µM
Archaea	F: CGGTGAATAYGYCCCTGC	16S ribosomal RNA	[4]	0.25 µM
	R: AAGGAGGTGATCCRGCCGCA	(16S rRNA), (10^3 copies/PCR), 173 bp	[4]	1.00 µM
	P: FAM-CTTGCACACACCGCCCGTC-BHQ1		[2]	0.10 µM
Bacteria	F: TGGAGCATGTGGTTTAATTCGA	16S ribosomal RNA	[5]	0.25 µM
	R: TGCGGGACTTAACCCAACA	(16S rRNA), (10^3 copies/PCR), 159 bp	[5]	0.10 µM
	P: FAM-CACGAGCTGACGACARCCATGCA-BHQ1		[5]	0.10 µM

[a] F, forward primer; R, reverse primer; P, probe; FAM, 6-carboxyfluorescein; BHQ1, Black Hole Quencher 1.

Supplemental Table 2. Correlation of abundances between tongue scrapings and subgingival samples

	Abundances			Tongue scraping and Subgingival plaque > 0	
	Tongue scraping	Subgingival plaque	r_{SP} (95% CI)	N	r_{SP} (95% CI)
P. gingivalis	0<br>(0; $1.5\cdot10^{-5}$)	0<br>(0; $1.9\cdot10^{-4}$)	0.63<br>(0.55-0.70)	57	0.25<br>(-0.01-0.48)
A. actinomycetemcomitans	0<br>(0; 0)	0<br>(0; $8.4\cdot10^{-5}$)	0.16<br>(0.03-0.28)	15	0.03<br>(-0.49-0.53)
F. nucleatum	0<br>(0; 0)	$2.0\cdot10^{-3}$<br>(0; $1.4\cdot10^{-2}$)	0.21<br>(0.09-0.33)	44	0.06<br>(-0.24-0.35)
S. sanguinis	0<br>(0; $3.8\cdot10^{-6}$)	0<br>(0; $2.1\cdot10^{-3}$)	0.56<br>(0.46-0.64)	72	0.17<br>(-0.07-0.38)
Archaea	429.8<br>(0; 656.2)	0<br>(0; 0)	-0.10<br>(-0.23-0.03)	26	0.25<br>(-0.15-0.58)
% *Archaea*	0.01<br>(0; 0.02)	0<br>(0; 0)	-0.01<br>(-0.14-0.12)	26	-0.33<br>(-0.64-0.06)
Bacteria	$2.6\cdot10^{6}$<br>($1.5\cdot10^{6}$; $4.0\cdot10^{6}$)	$1.7\cdot10^{4}$<br>($9.9\cdot10^{3}$; $2.9\cdot10^{4}$)	-0.14<br>(-0.26-0.01)	237	-0.14<br>(-0.26- -0.01)

Abundances are given as Median (25% quantile, 75% quantile). r_{SP}, Spearman's correlation coefficient; CI, confidence interval

Supplemental Table 3. Median relative abundances (log10 transformed; with 25% and 75% quantiles) from subgingival pocket samples according to categories of mesiobuccal probing depths and clinical attachment levels (categorized as ≤2, 3 and ≥4 mm).

	P. gingivalis	*A. actinomy-cetemcomitans*	*F. nucleatum*	*S. sanguinis*	*Archaea*	% *Archaea*	Total bacteria	Sum score
mesiobuccal PD								
≤2 mm (N=46)	0 (0; $3.2\cdot10^{-5}$)	0 (0; 0)	$3.6\cdot10^{-4}$ (0; $2.6\cdot10^{-3}$)	0 (0; $1.2\cdot10^{-3}$)	0.00 (0.00; 0.00)	0 (0; 0)	4.18 (3.97; 4.36)	$1.7\cdot10^{-3}$ ($1.9\cdot10^{-4}$; $9.9\cdot10^{-3}$)
3 mm (N=130)	0 (0; $3.7\cdot10^{-5}$)	0 (0; $2.1\cdot10^{-5}$)	$9.3\cdot10^{-4}$ (0; $7.3\cdot10^{-3}$)	0 (0; $8.8\cdot10^{-4}$)	0.00 (0.00; 0.00)	0 (0; 0)	4.22 (4.00; 4.47)	$3.0\cdot10^{-3}$ ($3.6\cdot10^{-4}$; $2.7\cdot10^{-2}$)
≥4 mm (N=56)	$6.3\cdot10^{-6}$ (0; $1.3\cdot10^{-3}$)*,**	0 (0; $9.5\cdot10^{-5}$)	$1.1\cdot10^{-3}$ (0; $5.2\cdot10^{-3}$)	0 (0; $1.0\cdot10^{-3}$)	0.00 (0.00; 0.00)	0 (0; 0)	4.28 (3.99; 4.70)	$3.5\cdot10^{-2}$ ($1.2\cdot10^{-2}$; $3.8\cdot10^{-2}$) *
mesiobuccal CAL								
≤2 mm (N=100)	0 (0; $1.4\cdot10^{-5}$)	0 (0; 0)	$7.0\cdot10^{-4}$ (0; $3.1\cdot10^{-3}$)	0 (0; $8.0\cdot10^{-4}$)	0 (0; 0)	0 (0; 0)	4.23 (3.99; 4.46)	$1.0\cdot10^{-3}$ ($8.2\cdot10^{-5}$; $7.2\cdot10^{-3}$)
3 mm (N=38)	$6.5\cdot10^{-7}$ (0; $7.4\cdot10^{-4}$) *	0 (0; $2.6\cdot10^{-5}$)	$6.0\cdot10^{-4}$ (0; $4.8\cdot10^{-3}$)	0 (0; $1.3\cdot10^{-3}$)	0 (0; 2.34) *	0 (0; 0.48) *	4.24 (4.05; 4.57)	$2.0\cdot10^{-3}$ ($2.6\cdot10^{-4}$; $6.1\cdot10^{-3}$)
≥4 mm (N=48)	$8.5\cdot10^{-6}$ (0; $1.2\cdot10^{-3}$) *	0 (0; $9.7\cdot10^{-5}$) *	$1.9\cdot10^{-3}$ ($3.0\cdot10^{-4}$; $6.9\cdot10^{-3}$) *	0 (0; $1.0\cdot10^{-3}$)	0 (0; 1.19) *	0 (0; 0.25)	4.33 (4.09; 4.75) *	$3.8\cdot10^{-3}$ ($6.2\cdot10^{-4}$; $3.2\cdot10^{-2}$) *

The Sum score was defined as the sum of relative abundances for *P. gingivalis* + *A. actinomycetemcomitans* + *F. nucleatum*. *,** P<0.05 in Mann-Whitney U tests (versus ≤2 mm or versus 3 mm, respectively); PD, probing depth; CAL, clinical attachment level

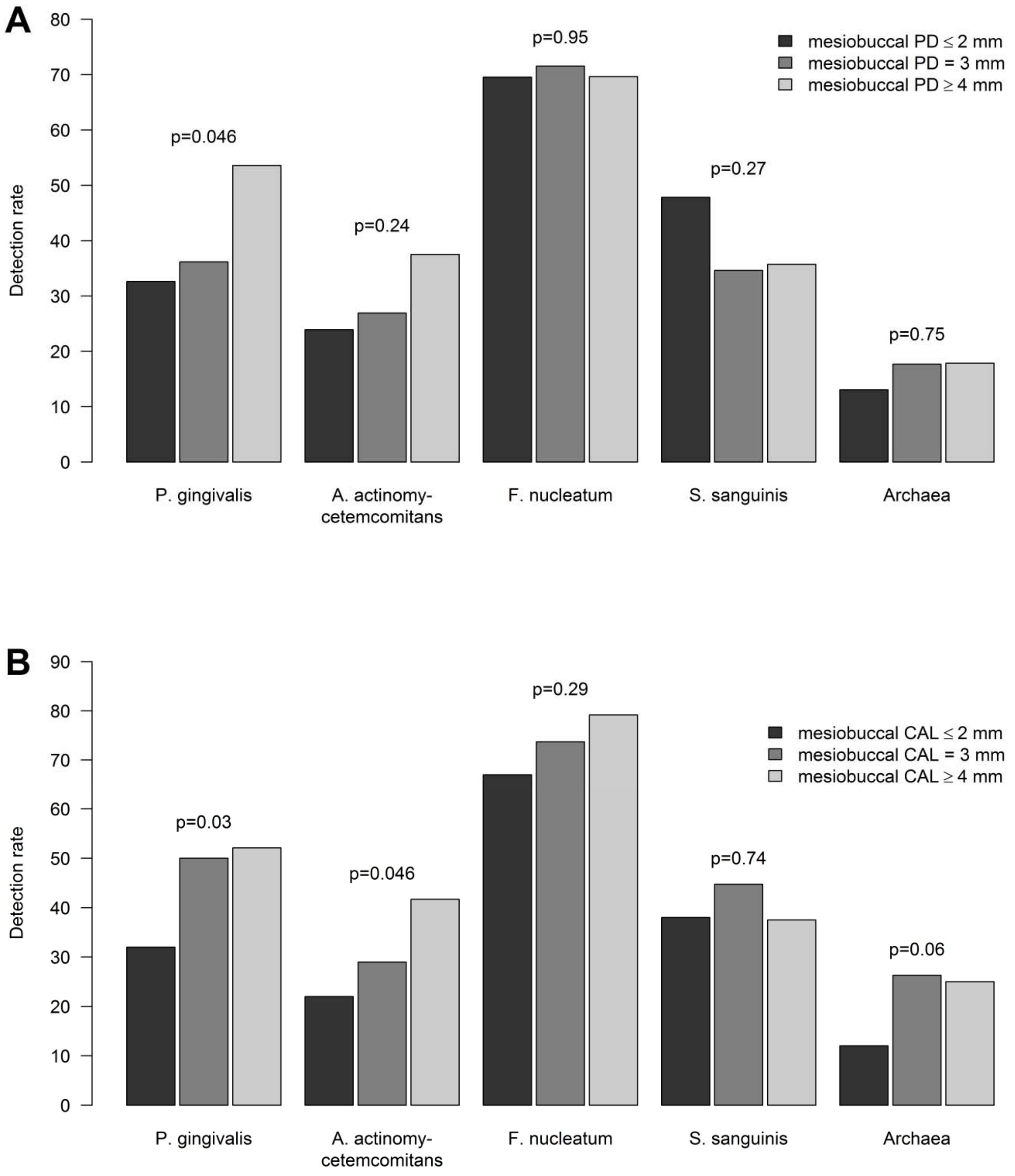

Supplemental Figure 1. Detection rates of bacteria in subgingival plaques according to (A) mesiobuccal probing depth (PD) or to (B) mesiobuccal clinical attachment level (CAL). P values were calculated from Chi squared tests evaluating differences in detection rates among groups.

REFERENCES

1. Morillo JM, Lau L, Sanz M, et al. Quantitative real-time polymerase chain reaction based on single copy gene sequence for detection of periodontal pathogens. J Clin Periodontol. 2004 Dec;31(12):1054-60. doi: CPE608 [pii] 10.1111/j.1600-051x.2004.00608.x. PubMed PMID: 15560805; eng.
2. Gohler A, Hetzer A, Holtfreter B, et al. Quantitative molecular detection of putative periodontal pathogens in clinically healthy and periodontally diseased subjects. PLoS One. 2014;9(7):e99244. doi: 10.1371/journal.pone.0099244 PONE-D-14-03721 [pii]. PubMed PMID: 25029268; PubMed Central PMCID: PMC4100758. eng.
3. Morikawa M, Chiba T, Tomii N, et al. Comparative analysis of putative periodontopathic bacteria by multiplex polymerase chain reaction. J Periodontal Res. 2008 Jun;43(3):268-74. doi: 10.1111/j.1600-0765.2007.01025.x JRE1025 [pii]. PubMed PMID: 18447853; eng.
4. Suzuki MT, Taylor LT, DeLong EF. Quantitative analysis of small-subunit rRNA genes in mixed microbial populations via 5'-nuclease assays. Appl Environ Microbiol. 2000 Nov;66(11):4605-14. PubMed PMID: 11055900; PubMed Central PMCID: PMC92356. eng.
5. Yang S, Lin S, Kelen GD, et al. Quantitative multiprobe PCR assay for simultaneous detection and identification to species level of bacterial pathogens. J Clin Microbiol. 2002 Sep;40(9):3449-54. PubMed PMID: 12202592; PubMed Central PMCID: PMC130696. eng.
